# Greater Trochanteric Pain Syndrome in Patients with Degenerative Lumbar Spine Disease: Clinical Findings and Short-Term Outcomes in a Retrospective Cohort Study

**DOI:** 10.3390/healthcare14183097

**Published:** 2026-09-20

**Authors:** Muhammet Kırkgeçit, Muhammet Erdi Gürbüz, Recai Engin, Hasan Türkoğlu, Fırat Yıldız, Muharrem Furkan Yüzbaşı, Emrullah Cem Kesilmez, Ersoy Kocabıçak

**Affiliations:** 1 Department of Neurosurgery, Megapoint Hospital, Kahramanmaraş 46100, Türkiye; mkirkgecit46@gmail.com; 2 Department of Orthopedics and Traumatology, HG Hospital, Kahramanmaraş 46100, Türkiye; 3 Department of Neurosurgery, Samsun University, Samsun 55080, Türkiye; 4 Department of Neurosurgery, Gaziantep City Hospital, Gaziantep 27470, Türkiye; 5 Department of Neurosurgery, Aksaray Training and Research Hospital, Aksaray 68100, Türkiye; 6 Department of Neurosurgery, Kahramanmaraş Sütçü İmam University, Kahramanmaraş 46050, Türkiye; 7 Department of Neurosurgery, Faculty of Medicine, Istanbul Atlas University, Istanbul 34408, Türkiye

**Keywords:** greater trochanteric pain syndrome, lumbar degenerative disease, extra-spinal pain, multidisciplinary assessment, corticosteroid injection, hip pain

## Abstract

**Background:** Greater trochanteric pain syndrome (GTPS) may coexist with lumbar degenerative spine disease (LDSD) and complicate pain attribution. **Methods:** This retrospective cohort study evaluated the observed frequency and short-term clinical course of GTPS in a selected tertiary-care LDSD population and explored factors associated with a previous surgical recommendation. Among 153 consecutive eligible patients with clinically compatible and MRI-documented LDSD, 63 had clinical suspicion of GTPS; all 63 underwent hip MRI, demonstrated findings consistent with trochanteric bursitis, received ultrasound-guided trochanteric bursa injection plus a two-week non-steroidal anti-inflammatory drug course, and completed one- and three-month follow-up. **Results:** GTPS-related lateral hip pain decreased from 7.65 ± 0.86 at baseline to 2.62 ± 0.68 at one month and 1.97 ± 1.43 at three months (Friedman *p* < 0.001; Kendall’s W = 0.896). Twenty-five patients (39.7%) had received an outside-center lumbar surgical recommendation before GTPS recognition. In exploratory multivariable analysis, each additional pre-diagnosis outpatient visit (OR 5.82; 95% CI 2.41–14.07) and prior lumbar surgery (OR 8.75; 95% CI 1.23–62.15) were associated with previous surgical recommendation. **Conclusions:** These findings support careful assessment of extra-spinal pain generators in LDSD, while the retrospective uncontrolled design precludes causal inference.

## 1. Introduction

Low-back pain is a leading global cause of disability [1]. Lumbar degenerative spine disease (LDSD) encompasses degenerative conditions such as intervertebral disc degeneration, lumbar spinal stenosis, and degenerative spondylolisthesis [2]. Degenerative lumbar MRI findings are also common in asymptomatic individuals, so radiological abnormalities require careful correlation with the clinical presentation [3,4]. Symptomatic LDSD contributes substantially to the global burden of low-back pain [5]. Degenerative change reflects interacting biomechanical, cellular, and inflammatory processes rather than aging alone [6,7,8,9].

Greater trochanteric pain syndrome (GTPS) is a common cause of lateral hip pain [10,11]. Population-based data demonstrate a clear female predominance and an association with low-back pain [12]. GTPS is primarily a clinical diagnosis, and characteristic lateral hip symptoms and examination findings are central to differentiating it from other causes of hip and lumbar-region pain [13,14]. Specific clinical tests can further support the diagnosis in appropriately selected patients [15].

Substantial overlap has been reported in spine-clinic populations: GTPS has been identified in approximately 20% to 50% of selected patients evaluated for lumbar pathology [16,17]. Conversely, lumbosacral pathology is also frequently documented among patients evaluated for GTPS [18]. This overlap can complicate pain attribution and may contribute to repeated spine-focused evaluation when extra-spinal pain generators are not considered.

GTPS is not synonymous with isolated trochanteric bursitis; abductor tendon pathology is an important component of the broader peritrochanteric spectrum [19]. Accordingly, careful clinical assessment is important before attributing lateral hip and thigh symptoms solely to degenerative lumbar abnormalities.

Failure to evaluate extra-spinal pain sources may delay diagnosis and complicate treatment strategy, particularly when surgical indications are being weighed. Early recognition of extra-spinal pain generators may facilitate appropriate referral and multidisciplinary assessment. Therefore, the present study aimed to determine the observed frequency of GTPS among patients with degenerative lumbar spine disease in this selected clinical cohort, characterize affected patients, evaluate short-term changes in GTPS-related lateral hip pain following treatment, and explore factors associated with a previous surgical recommendation before GTPS diagnosis.

## 2. Materials and Methods

### 2.1. Study Design and Ethical Approval

This was a retrospective cohort study conducted at a tertiary care center across neurosurgery and orthopedics outpatient clinics. The protocol was approved by the Kahramanmaraş Sütçü İmam University Clinical Research Ethics Committee (Approval No: 67), and all procedures were carried out in accordance with the principles of the Declaration of Helsinki. Ethics committee approval was obtained for the retrospective analysis of previously collected clinical data after completion of the clinical observation period; no prospective study intervention was performed under this approval.

### 2.2. Patient Selection and Eligibility

Between January 2025 and January 2026, 153 consecutive eligible patients presenting to neurosurgery and orthopedics outpatient clinics were retrospectively reviewed. LDSD was operationally defined as clinically compatible low-back and/or lower-extremity symptoms together with one or more degenerative abnormalities on lumbar MRI, including intervertebral disc degeneration or disc pathology, central and/or foraminal lumbar stenosis, and/or degenerative spondylolisthesis [2,5]. Imaging abnormalities were interpreted in conjunction with the clinical presentation rather than in isolation. Patients with trauma, infection, malignancy, inflammatory rheumatological disease, or incomplete records were not eligible for the screening cohort. All 153 patients underwent routine clinical assessment for hip and peritrochanteric symptoms. Hip MRI was selectively obtained in the 63 patients with clinical suspicion of GTPS; all 63 demonstrated MRI findings consistent with trochanteric bursitis and formed the analytic cohort. No negative or inconclusive hip MRI examinations occurred in this clinically selected subgroup. All 63 had documented one- and three-month VAS assessments, with no loss to follow-up. Detailed LDSD imaging subcategories were not systematically coded for comparative analysis.

The remaining 90 patients did not enter the GTPS outcome analyses and were not used as a formal GTPS-negative comparison group. Consequently, the observed 63/153 proportion describes the frequency of the selected clinical phenotype identified through this routine-care pathway and should not be interpreted as the population prevalence of the full GTPS spectrum.

### 2.3. Diagnosis of GTPS

**Figure 1:**
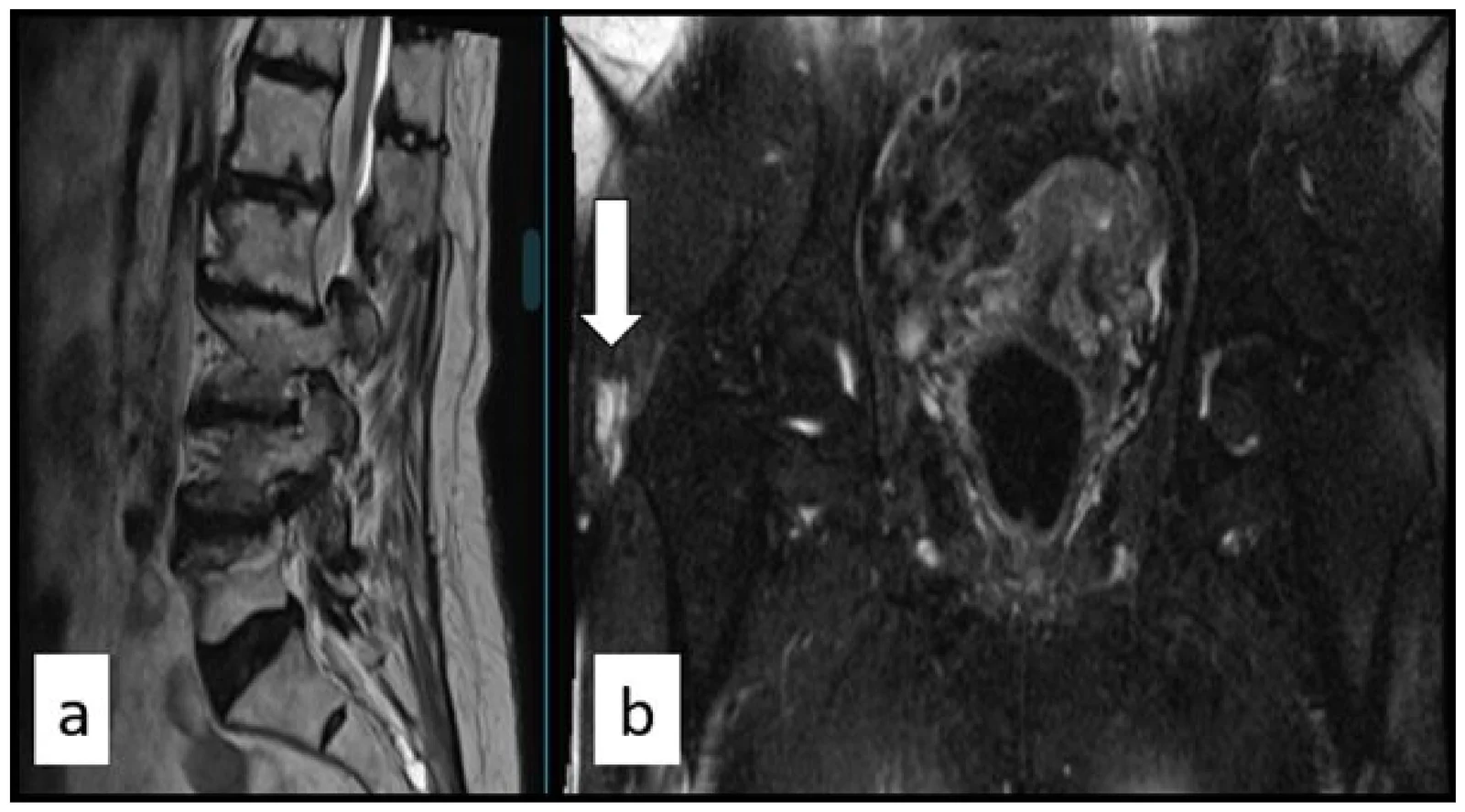
Representative anonymized MRI images from two different participants. (**a**) Sagittal T2-weighted lumbar MRI demonstrating degenerative spondylosis with right-sided foraminal stenosis at L3–4 and L4–5. (**b**) Coronal T2-weighted hip MRI from a different participant demonstrating right peritrochanteric hyperintensity consistent with trochanteric bursitis (white arrow). The panels are representative examples and were not used for patient-level lumbar–hip laterality analysis.

All 153 patients underwent routine neurological and musculoskeletal examination, including hip assessment as part of the evaluation of degenerative lumbar spine disease. Clinical suspicion of GTPS was based on characteristic lateral hip pain together with a compatible combination of examination findings, including focal tenderness over the greater trochanter and reproduction of lateral hip pain during resisted hip abduction; pain while lying on the affected side was considered a supportive historical feature [10,13,14,15,20,21]. Because this was a retrospective study reflecting routine clinical practice, a rigid requirement for all findings to be simultaneously positive was not prospectively defined. Alternative hip and pelvic pain generators, including intra-articular hip and sacroiliac joint-related pain, were considered during routine differential clinical assessment. These examination components were part of usual care but were not prospectively recorded as separate study variables. Representative lumbar and hip MRI findings are shown in Figure 1.

Hip MRI was selectively obtained when GTPS was clinically suspected and was used to characterize associated peritrochanteric pathology rather than as a universal screening test or a stand-alone diagnostic criterion. MRI findings considered consistent with trochanteric bursitis included fluid distension of the trochanteric bursa and/or increased fluid-sensitive signal within the peritrochanteric bursal region, as documented in the contemporaneous radiology report [21]. All 63 patients in the analytic cohort had MRI findings consistent with trochanteric bursitis. The same radiologist evaluated the hip MRI examinations and, because imaging was obtained as part of routine clinical care, was aware of the relevant clinical history and was not blinded to the clinical suspicion of GTPS. Gluteus medius/minimus tendinopathy was not systematically graded or recorded and therefore could not be reliably analyzed. Accordingly, the analytic cohort represents clinically suspected GTPS with MRI evidence of trochanteric bursitis rather than the entire pathoanatomical spectrum of GTPS.

### 2.4. Treatment Protocol

**Figure 2:**
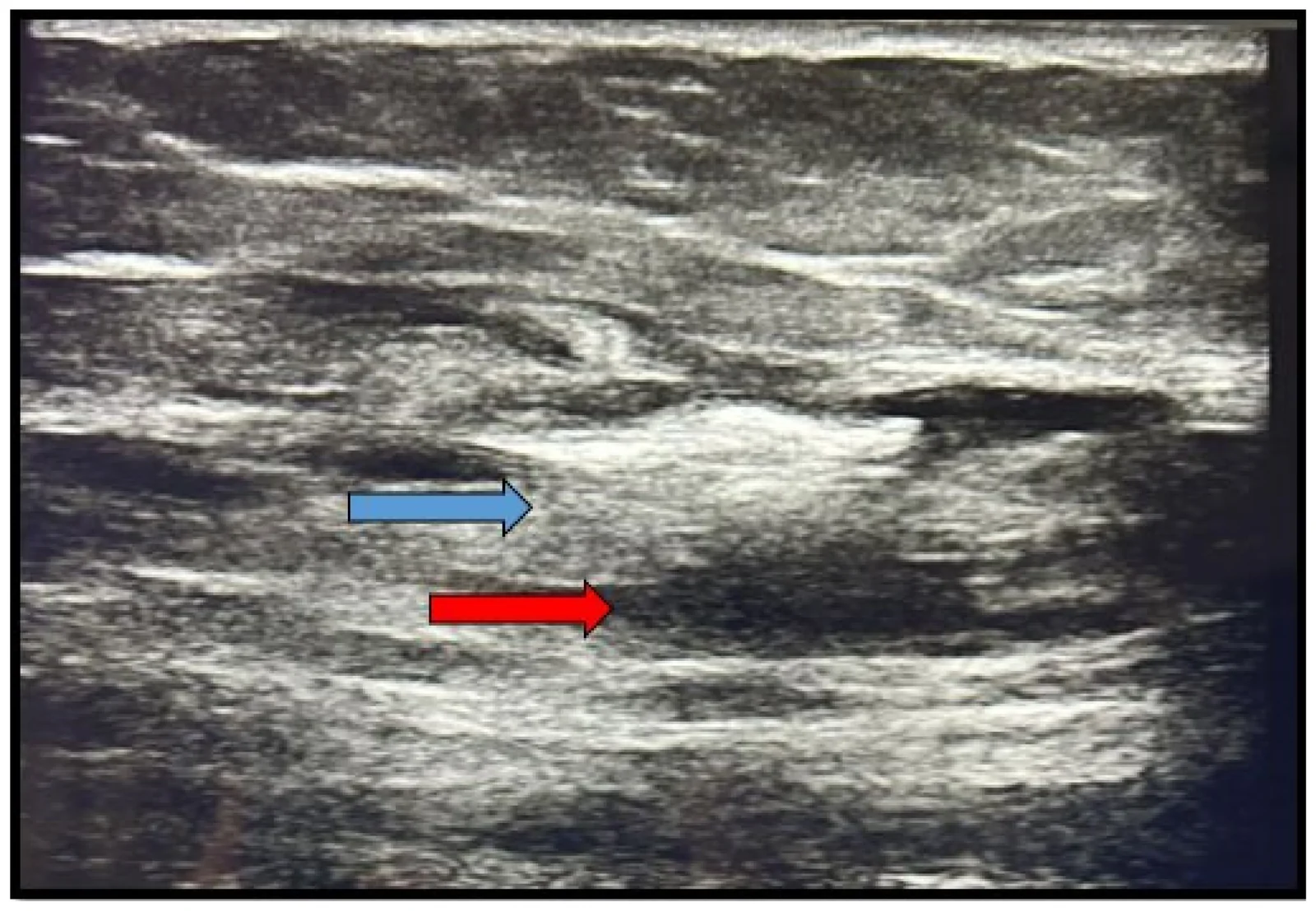
Ultrasound-guided trochanteric bursa injection. The needle is indicated by the blue arrow, and the hypoechoic area representing the trochanteric bursa is indicated by the red arrow. This is a representative anonymized image from a patient included in the study cohort.

All 63 patients in the analytic GTPS cohort received ultrasound-guided trochanteric bursa injection (Figure 2).

Ultrasound was used here to guide the therapeutic procedure, not for diagnostic purposes. Patients were positioned in lateral decubitus under sterile conditions; a linear transducer was used to visualize the greater trochanter and surrounding soft tissues, and a 22-gauge needle was advanced into the bursa. Each patient received a 5 mL injection consisting of 1 mL of a betamethasone preparation containing 5 mg betamethasone dipropionate and 2 mg betamethasone sodium phosphate combined with 4 mL of 0.5% bupivacaine. All procedures were performed by the same orthopedic physician using the same ultrasound-guided technique. All patients also received a 2-week course of NSAID therapy. The specific NSAID agent and dosage were individualized according to patient age, comorbidities, and clinical considerations; therefore, a standardized NSAID regimen was not used.

### 2.5. Clinical Assessment and Outcome Measures

Pain severity was measured using the Visual Analog Scale (VAS; 0–10) at baseline, one month, and three months after the procedure. VAS specifically assessed lateral hip pain attributed to GTPS and did not represent low-back pain. All VAS assessments were recorded by the same physician, who was not blinded to the patients’ clinical information or treatment status. The primary outcome was change in GTPS-related lateral hip pain VAS over time. Secondary variables included the number of neurosurgical outpatient visits before GTPS diagnosis, history of prior spinal surgery, previous surgical recommendation, and presence of neurological deficit. The variable “previous surgical recommendation” referred to a recommendation for lumbar spine surgery made at an outside center because of persistent symptoms before GTPS was recognized in the present evaluation. Thus, outpatient visit frequency and previous surgical recommendation preceded GTPS diagnosis and treatment and were not post-treatment outcomes.

### 2.6. Statistical Analysis

Analyses were performed using IBM SPSS Statistics Version 25.0. Continuous variables are reported as mean ± standard deviation (SD) with minimum–maximum values and, for key non-normally distributed variables, median [interquartile range (IQR)]; categorical variables as counts and percentages. Normality was assessed with the Kolmogorov––Smirnov and Shapiro––Wilk tests; variance homogeneity was evaluated with Levene’s test. The Shapiro––Wilk test rejected the normality assumption for all continuous variables (*p* < 0.05), and Levene’s test indicated heterogeneous variances; the Mann––Whitney U test was therefore used for between-group comparisons.

Because repeated VAS measurements were non-normally distributed, within-patient changes across baseline, 1-month, and 3-month assessments were analyzed with the Friedman test. When the omnibus test was significant, pairwise comparisons were conducted using Bonferroni-corrected Wilcoxon signed-rank tests (significance threshold α = 0.017; i.e., 0.05/3 for three pairwise comparisons), with differences reported as paired median reductions between time points. Categorical variables were compared using chi-square or Fisher’s exact test when expected cell counts fell below five. Effect sizes for non-parametric comparisons were calculated as r = |z|/√*N*. Spearman correlation was used to assess relationships among continuous variables. Statistical significance was set at *p* < 0.05 unless otherwise specified.

**Figure 3:**
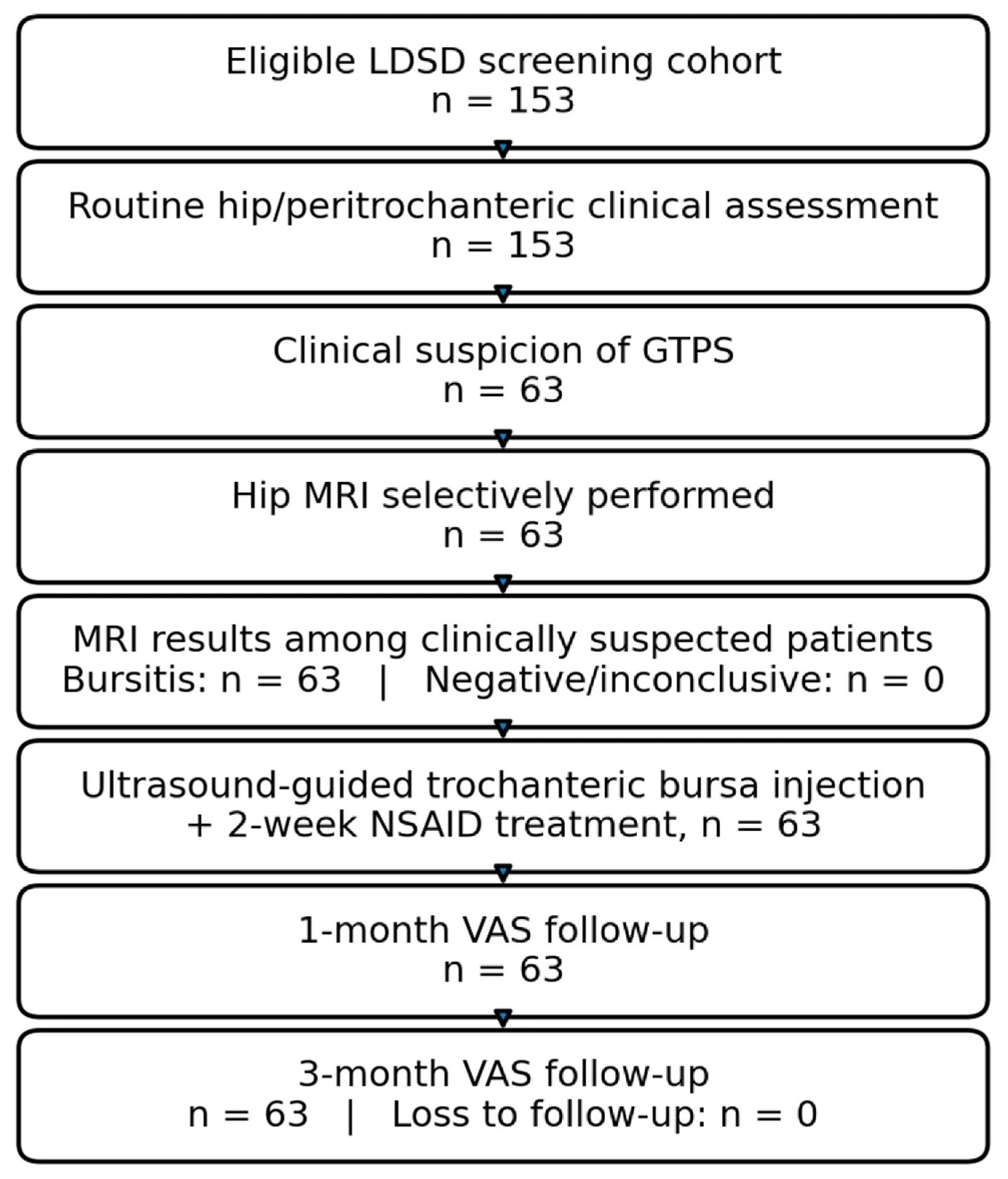
Patient flow and clinical timeline. Eligible LDSD screening cohort, *n* = 153; routine hip/peritrochanteric clinical assessment, *n* = 153; clinical suspicion of GTPS, *n* = 63; hip MRI, *n* = 63; MRI findings consistent with trochanteric bursitis, *n* = 63; negative/inconclusive MRI, *n* = 0; ultrasound-guided trochanteric bursa injection plus 2-week NSAID treatment, *n* = 63; 1-month VAS follow-up, *n* = 63; 3-month VAS follow-up, *n* = 63; loss to follow-up, *n* = 0. Previous neurosurgical visits and outside-center lumbar surgical recommendations occurred before GTPS recognition.

An exploratory multivariable logistic regression analysis was performed to examine associations between selected clinical variables and a previous surgical recommendation before GTPS diagnosis. Variables with *p* < 0.10 on univariate analysis were entered into the model; these were baseline VAS score, number of pre-diagnosis outpatient visits, and history of previous lumbar spine surgery. Results are reported as adjusted odds ratios (OR) with 95% confidence intervals (CI). Given the limited number of patients with a previous surgical recommendation and the sparse distribution of prior surgery, the regression results were interpreted cautiously as exploratory associations rather than definitive independent predictors. Model fit was assessed with Nagelkerke R^2^ and the Hosmer-Lemeshow goodness-of-fit test. The patient flow and clinical timeline are shown in Figure 3.

## 3. Results

### 3.1. General Findings and VAS Score Changes

Of the 153 eligible patients with LDSD in the screening cohort, 63 had clinical suspicion of GTPS, underwent hip MRI, and demonstrated MRI findings consistent with trochanteric bursitis; these 63 patients formed the analytic cohort (41.1%; 95% Wilson CI: 33.7–49.1%). This proportion represents the observed frequency of this selected clinical phenotype and should not be interpreted as a population prevalence estimate for the full GTPS spectrum. All 63 patients completed the one- and three-month follow-up assessments. Mean age was 61.13 ± 11.46 years (median 61 [IQR 51–72]), and the mean number of pre-diagnosis neurosurgical outpatient visits was 2.25 ± 0.84 (median 2 [IQR 2–3]). GTPS-related lateral hip pain VAS decreased from 7.65 ± 0.86 (median 8 [IQR 7–8]) at baseline to 2.62 ± 0.68 (median 3 [IQR 2–3]) at one month and 1.97 ± 1.43 (median 2 [IQR 1–2]) at three months. The overall change was significant (Friedman χ^2^ = 112.84; *p* < 0.001; Kendall’s W = 0.896). Bonferroni-corrected Wilcoxon tests showed median within-patient reductions of 5 points from baseline to month 1 (z = −6.99; r = 0.88), 1 point from month 1 to month 3 (z = −3.87; r = 0.49), and 6 points from baseline to month 3 (z = −6.95; r = 0.88) (all *p* < 0.001).

### 3.2. Demographic and Clinical Characteristics

Seventy-three percent of patients were women. The predominant presentation was combined low back and hip pain, accounting for 92.1% of cases; the remaining 5 patients (7.9%) reported hip pain alone. Prior spinal surgery was documented in 6 patients (9.5%). Neurological deficit was present in 2 patients (3.2%) (Table 1). Before GTPS diagnosis in the present evaluation, 25 patients (39.7%) had previously received a recommendation for lumbar spine surgery at an outside center because of persistent symptoms, whereas 38 (60.3%) had not.

### 3.3. Surgical History, Previous Surgical Recommendation, and VAS Score Comparisons

Patients with a history of prior spinal surgery had received a previous surgical recommendation at a higher rate: 5 of 6 patients (83.3%) versus 20 of 57 (35.1%) without prior surgery (*p* = 0.032). Patients with prior surgery also had more pre-diagnosis outpatient visits (3.33 ± 0.52; median 3 [IQR 3–3.75] vs. 2.14 ± 0.79; median 2 [IQR 2–3]; *p* = 0.001; r = 0.40). Patients with a previous surgical recommendation had more pre-diagnosis outpatient visits (3.04 ± 0.54; median 3 [IQR 3–3] vs. 1.74 ± 0.55; median 2 [IQR 1–2]; *p* < 0.001; r = 0.78) and higher baseline VAS scores (7.96 ± 0.79; median 8 [IQR 8–8] vs. 7.45 ± 0.86; median 7 [IQR 7–8]; *p* = 0.016; r = 0.30). VAS scores at one and three months were numerically higher in the previous-recommendation group, but the between-group differences were not statistically significant (*p* = 0.369 and *p* = 0.287, respectively). Women had higher baseline VAS scores than men (7.78 ± 0.87; median 8 [IQR 7–8] vs. 7.29 ± 0.77; median 7 [IQR 7–8]; *p* = 0.042; r = 0.26) (Table 2).

### 3.4. Exploratory Associations with Previous Surgical Recommendation: Multivariable Logistic Regression

Variables meeting the prespecified *p* < 0.10 threshold on univariate analysis—pre-diagnosis outpatient visit frequency, baseline VAS score, and prior surgical history—were entered into the exploratory multivariable logistic regression model. Model fit was Nagelkerke R^2^ = 0.527 with Hosmer-Lemeshow *p* = 0.643. Each additional pre-diagnosis outpatient visit was associated with higher odds of a previous surgical recommendation (OR: 5.82 per additional visit; 95% CI: 2.41–14.07; *p* < 0.001), and prior spinal surgery was also associated with higher odds (OR: 8.75; 95% CI: 1.23–62.15; *p* = 0.028). Baseline VAS did not reach statistical significance after adjustment (OR: 2.14; 95% CI: 0.87–5.26; *p* = 0.098). Given only 25 recommendation events and six patients with prior spinal surgery, these estimates are statistically fragile and should be interpreted as exploratory retrospective associations rather than predictive effects. The regression results are presented in Table 3.

### 3.5. Correlations Among Continuous Variables: Spearman Analysis

Baseline VAS score and pre-diagnosis outpatient visit frequency showed a moderate positive correlation (ρ = 0.38; *p* = 0.002), indicating an association between greater pain severity at presentation and more frequent pre-diagnosis clinic attendance; this correlation should not be interpreted causally. The association between age and visit frequency was not statistically significant (ρ = 0.23; *p* = 0.068), and no significant association was found between age and baseline VAS (ρ = 0.19; *p* = 0.132) (Table 4).

## 4. Discussion

In this selected tertiary-care cohort of symptomatic patients with LDSD, 63 of 153 patients (41.1%; 95% CI, 33.7–49.1%) had clinically suspected GTPS with MRI findings consistent with trochanteric bursitis and formed the analytic cohort. GTPS-related lateral hip pain decreased substantially during three months of follow-up after ultrasound-guided injection combined with a 2-week NSAID course. Because there was no untreated or alternative-treatment comparison group, this temporal improvement cannot be attributed specifically to the injection. Greater pre-diagnosis outpatient utilization and prior spinal surgery were associated with a surgical recommendation that had been made before GTPS recognition; these findings describe retrospective associations and do not demonstrate that GTPS caused referral or that its treatment reversed surgical decision-making.

The observed 41.1% proportion should be interpreted in the context of the study pathway rather than as a population prevalence estimate. Hip MRI was selectively performed only in clinically suspected cases, and the analytic cohort required MRI evidence of trochanteric bursitis. Previous spine-clinic studies have nevertheless documented substantial overlap: Tortolani et al. reported GTPS in 20.2% of patients referred to a tertiary spine center, whereas Tan et al. identified GTPS in 50.5% of patients evaluated for degenerative lumbar pathology [16,17]. Lumbosacral pathology is also frequently documented among patients evaluated for GTPS [18]. These findings support considering GTPS during evaluation of selected patients with lumbar pathology but do not justify generalization to unselected populations.

The symptom pattern further illustrates the diagnostic overlap: 92.1% of the analytic cohort reported both low-back and hip pain, whereas only 7.9% reported hip pain alone. Tortolani et al. described GTPS as a syndrome capable of mimicking lumbar nerve-root symptoms and found objective neurological findings in only a minority of affected patients [17]. In our cohort, neurological deficit was present in 3.2%. This low frequency does not establish that the predominant pain burden was extra-spinal; rather, it underscores the diagnostic complexity of pain attribution in patients with degenerative lumbar imaging findings and the importance of evaluating potential extra-spinal pain generators.

Pain reduction during follow-up was substantial. VAS fell from 7.65 at baseline to 2.62 at one month and 1.97 at three months (Kendall’s W = 0.896), with most of the observed reduction occurring in the first month. Long-term observational data indicate that GTPS may persist in a substantial proportion of patients [22]. A 2022 meta-analysis of eight randomized trials concluded that evidence for corticosteroid injection in GTPS remains equivocal: injection may be superior to usual care or wait-and-see in some short- to medium-term comparisons, but it was not consistently superior to exercise or platelet-rich plasma [23]. Mellor et al. reported greater 8-week improvement after corticosteroid injection than wait-and-see, while education plus exercise produced better longer-term global improvement [24]. Because all patients in our cohort also received a two-week individualized NSAID course and there was no untreated or alternative-treatment control group, the observed pain reduction cannot be attributed solely to the injection.

The temporal definition of the surgical variable is important. The 25 surgical recommendations were made at outside centers before GTPS was recognized in the present evaluation, and outpatient visit frequency likewise represented visits accumulated before GTPS recognition. Therefore, the study does not evaluate whether GTPS treatment causedcancelation, postponement, continuation, or initiation of a surgical recommendation. The association between repeated spine-care encounters and a previous surgical recommendation is clinically relevant as a marker of diagnostic complexity, but whether systematic GTPS assessment changes operative decision-making requires prospective comparative study [16,17].

The exploratory multivariable model showed that each additional pre-diagnosis outpatient visit was associated with higher odds of a previous surgical recommendation (OR: 5.82 per additional visit; 95% CI: 2.41–14.07; *p* < 0.001), and prior spinal surgery was also associated with higher odds (OR: 8.75; 95% CI: 1.23–62.15; *p* = 0.028). Baseline VAS was not statistically significant after adjustment. Because only 25 recommendation events occurred and only six patients had prior spinal surgery, the estimates—particularly the wide CI for prior surgery—are unstable and hypothesis-generating. These results should not be interpreted as a predictive model or as evidence of a causal pathway.

Prior spine surgery may identify a clinically complex subgroup in whom persistent symptoms prompt repeated reassessment. WFNS recommendations for recurrent lumbar disc herniation emphasize individualized treatment selection and do not recommend routine fusion for all recurrent cases [25]. However, our dataset cannot establish whether GTPS was present before earlier operations, whether it was considered when outside surgical recommendations were made, or whether recognition of GTPS would have changed those decisions. Any suggestion that earlier GTPS recognition could reduce premature escalation of spine-directed treatment therefore remains a hypothesis for prospective investigation.

Women comprised 73.0% of the analytic cohort and had a modestly higher baseline VAS than men (7.78 vs. 7.29; *p* = 0.042; r = 0.26). Female predominance is consistent with population-based GTPS epidemiology and spine-clinic series [12,16,17]. We do not interpret the observed sex difference in pain severity as mechanistically explanatory because of the modest effect size and unbalanced sex distribution.

From a broader musculoskeletal-care perspective, early recognition of GTPS may have value beyond differential diagnosis alone. Assessment of both spinal and extra-spinal pain generators can facilitate appropriate referral and coordinated management involving spine specialists, orthopedic clinicians, radiologists, and rehabilitation professionals. Such an integrated approach may support earlier targeted treatment and more comprehensive recovery while avoiding premature escalation of spine-directed management.

This study has several limitations. First, its retrospective, single-center design and selected tertiary-care population introduce selection bias and limit generalizability. Hip MRI was selectively obtained only in patients with clinical suspicion of GTPS, and inclusion in the analytic cohort required MRI findings consistent with trochanteric bursitis. The study may therefore preferentially represent a narrower GTPS phenotype and may under-recognize clinically relevant GTPS dominated by gluteus medius/minimus tendinopathy without bursal abnormalities; the observed 41.1% frequency should not be interpreted as the prevalence of the full GTPS spectrum. Second, the remaining 90 patients were not followed as a formal GTPS-negative comparison group, preventing causal comparisons according to GTPS status. Third, detailed LDSD imaging subcategories, GTPS laterality relative to lumbar pathology, gluteal tendon status, and the individual components of the routine hip/sacroiliac differential examination were not systematically coded for comparative or mechanistic analysis. Hip MRI was interpreted in the routine-care setting with the relevant clinical history available to the radiologist rather than under study-specific blinded conditions. Fourth, information on whether previous surgical recommendations were subsequently maintained, deferred, withdrawn, or followed by surgery was unavailable. Fifth, serial VAS measurements assessed GTPS-related lateral hip pain only; longitudinal low-back-pain VAS and validated functional measures such as ODI or LEFS were unavailable. Sixth, all patients received trochanteric injection together with an individualized two-week NSAID course, so the independent effect of injection cannot be isolated. Seventh, VAS follow-up was performed by the same treating physician without blinding. Finally, the exploratory regression included only 25 recommendation events and six patients with prior lumbar surgery, resulting in limited model stability and imprecise estimates.

## 5. Conclusions

In this selected tertiary-care LDSD cohort, clinically suspected GTPS accompanied by MRI findings consistent with trochanteric bursitis was frequently identified. GTPS-related lateral hip pain decreased substantially during three months of follow-up after ultrasound-guided trochanteric injection plus short-term NSAID therapy, although the uncontrolled retrospective design precludes causal attribution to the injection itself. More frequent pre-diagnosis spine-clinic visits and prior lumbar surgery were retrospectively associated with a previous outside-center surgical recommendation, but these exploratory associations do not demonstrate that GTPS caused referral or that its treatment altered surgical decisions. Careful assessment for extra-spinal pain generators may improve diagnostic characterization in patients with LDSD; whether systematic GTPS assessment changes subsequent spine-related treatment decisions should be tested prospectively.

## Figures and Tables

**Table 1:** Demographic, clinical characteristics and VAS score changes.

Variable	Value	Statistic/*p* Value
Age (years), mean ± SD [min–max]	61.13 ± 11.46 [37–85]; median 61 [51–72]	—
Outpatient visits, mean ± SD [min–max]	2.25 ± 0.84 [1–4]; median 2 [2–3]	—
VAS (baseline), mean ± SD [min–max]	7.65 ± 0.86 [6–9]; median 8 [7–8]	Friedman: χ^2^ = 112.84; *p* < 0.001; Kendall’s W = 0.896
VAS (month 1), mean ± SD [min–max]	2.62 ± 0.68 [1–4]; median 3 [2–3]	—
VAS (month 3), mean ± SD [min–max]	1.97 ± 1.43 [1–7]; median 2 [1–2]	—
**Post hoc: baseline → month 1, paired median reduction (*p* †)**	5.00	*p* < 0.001; z = −6.99; r = 0.88
**Post hoc: month 1 → month 3, paired median reduction (*p* †)**	1.00	*p* < 0.001; z = −3.87; r = 0.49
**Post hoc: baseline → month 3, paired median reduction (*p* †)**	6.00	*p* < 0.001; z = −6.95; r = 0.88
Female sex, *n* (%)	46 (73.0%)	—
Male sex, *n* (%)	17 (27.0%)	—
Complaint: low back + hip pain, *n* (%)	58 (92.1%)	—
Complaint: hip pain only, *n* (%)	5 (7.9%)	—
Prior spinal surgery, *n* (%)	6 (9.5%)	—
No prior spinal surgery, *n* (%)	57 (90.5%)	—
Previous surgical recommendation before GTPS diagnosis, *n* (%)	25 (39.7%)	—
No previous surgical recommendation before GTPS diagnosis, *n* (%)	38 (60.3%)	—
Neurological deficit present, *n* (%)	2 (3.2%)	—
Neurological deficit absent, *n* (%)	61 (96.8%)	—

Continuous variables are expressed as mean ± SD [min–max] and, for key non-normally distributed variables, median [IQR]. Friedman test applied for repeated VAS measurements. † Bonferroni-corrected Wilcoxon signed-rank test (3 pairwise comparisons; significance threshold α = 0.017). Pairwise results are reported as median within-patient reductions with z statistics and effect sizes (r = |z|/√*N*). Categorical variables are expressed as *n* (%). VAS: Visual Analog Scale; SD: standard deviation; IQR: interquartile range.

**Table 2:** Between-group clinical comparisons.

Variable—Group	*n*	Value (Mean ± SD; Median [IQR] or *n* [%])	*p* Value	Effect Size (r)
Previous surgical recommendation—prior surgery	6	5 (83.3%)	0.032 †	—
**No previous surgical recommendation—prior surgery**	6	1 (16.7%)		
Previous surgical recommendation—no prior surgery	57	20 (35.1%)		
**No previous surgical recommendation—no prior surgery**	57	37 (64.9%)		
**Pre-diagnosis outpatient visits—no prior surgery**	57	2.14 ± 0.79; 2 [2–3]	0.001	0.40
**Pre-diagnosis outpatient visits—prior surgery**	6	3.33 ± 0.52; 3 [3–3.75]		
**Pre-diagnosis outpatient visits—not previous recommendation**	38	1.74 ± 0.55; 2 [1–2]	<0.001	0.78
**Pre-diagnosis outpatient visits—previous recommendation**	25	3.04 ± 0.54; 3 [3–3]		
**VAS baseline—not previous recommendation**	38	7.45 ± 0.86; 7 [7–8]	0.016	0.30
**VAS baseline—previous recommendation**	25	7.96 ± 0.79; 8 [8–8]		
**VAS month 1—not previous recommendation**	38	2.55 ± 0.69; 2.5 [2–3]	0.369	0.11
**VAS month 1—previous recommendation**	25	2.72 ± 0.68; 3 [2–3]		
**VAS month 3—not previous recommendation**	38	1.82 ± 1.31; 1 [1–2]	0.287	0.13
**VAS month 3—previous recommendation**	25	2.20 ± 1.58; 2 [1–2]		
**VAS baseline—male**	17	7.29 ± 0.77; 7 [7–8]	0.042	0.26
**VAS baseline—female**	46	7.78 ± 0.87; 8 [7–8]		

Mann–Whitney U test applied for continuous variables. Continuous subgroup values are reported as mean ± SD and median [IQR]. † Fisher’s exact test compares previous surgical recommendation status between patients with and without prior surgery. *p* values and effect sizes (r = |z|/√*N*) are reported in the first row of each continuous-variable comparison. *p* < 0.05 was considered statistically significant. VAS: Visual Analog Scale; SD: standard deviation; IQR: interquartile range.

**Table 3:** Exploratory multivariable logistic regression of factors associated with previous surgical recommendation.

Variable	Wald	OR	95% CI (Lower)	95% CI (Upper)	*p* Value
Outpatient visit frequency	15.31	5.82	2.41	14.07	<0.001
Prior spinal surgery (yes)	4.91	8.75	1.23	62.15	0.028
VAS (baseline)	2.73	2.14	0.87	5.26	0.098
Constant	11.23	—	—	—	0.001

Dependent variable: previous surgical recommendation before GTPS diagnosis (yes/no). Total analysis *N* = 63; previous surgical recommendation events *n* = 25. For outpatient visit frequency, the OR is expressed per one additional pre-diagnosis visit. Model fit: Nagelkerke R^2^ = 0.527; Hosmer-Lemeshow *p* = 0.643. OR: odds ratio; CI: confidence interval; VAS: Visual Analog Scale. Estimates are exploratory because of the limited event count and sparse prior-surgery subgroup.

**Table 4:** Spearman correlation analysis among continuous variables.

Variable Pair	ρ (Spearman)	*p* Value	Interpretation
VAS (baseline)—outpatient visit frequency	0.38	0.002	Moderate positive correlation
Age—outpatient visit frequency	0.23	0.068	Not statistically significant
Age—VAS (baseline)	0.19	0.132	Not statistically significant

ρ: Spearman correlation coefficient. *p* < 0.05 is considered statistically significant. VAS: Visual Analog Scale.

## Data Availability

The raw data supporting the conclusions of this article will be made available by the authors on request.
